# Long-acting HIV Treatments: Study Design, Logistics, and Access

**DOI:** 10.1093/ofid/ofae337

**Published:** 2024-06-15

**Authors:** Nicholas A Murdock, Nayri E Alajaji, Robin Schaefer, Cheriko A Boone, Rafael E Campo, Gregory J Dore, Monica Gandhi, J Rafael Gorospe, Roy M Gulick, Sally L Hodder, Jonathan Liu, Martin S Rhee, James F Rooney, Vani Vannappagari, Timothy Wilkin, Veronica Miller

**Affiliations:** Forum for Collaborative Research, University of California, Berkeley, Washington, DC, USA; Forum for Collaborative Research, University of California, Berkeley, Washington, DC, USA; Forum for Collaborative Research, University of California, Berkeley, Washington, DC, USA; TAG Treatment Action Group Inc., New York, New York, USA; Merck & Co., Inc., Upper Gwynedd, Pennsylvania, USA; The Kirby Institute, University of New South Wales, Sydney, NSW, Australia; Department of Medicine, University of California at San Francisco (UCSF), San Francisco, California, USA; Office of AIDS Research, National Institutes of Health, Bethesda, Maryland, USA; Department of Medicine, Weill Cornell Medicine, New York, New York, USA; West Virginia Clinical and Translational Science Institute, West Virginia University School of Medicine, Morgantown, West Virginia, USA; University of Southern California, Los Angeles, California, USA; Gilead Sciences, Foster City, California, USA; Gilead Sciences, Foster City, California, USA; ViiV Healthcare, Durham, North Carolina, USA; Department of Medicine, Weill Cornell Medicine, New York, New York, USA; Forum for Collaborative Research, University of California, Berkeley, Washington, DC, USA

**Keywords:** HIV-1, inclusivity, long-acting HIV treatment, marginalized populations, trial design

## Abstract

New long-acting HIV treatment products have the potential to change the HIV epidemic in the United States and globally. Phase 3 clinical trials of HIV treatments tend to underrepresent populations bearing a disproportionate burden of the HIV epidemic—including women, racial minorities, trans and gender-diverse people, older adults, the unhoused, people who inject drugs, those in rural areas, individuals with mental illness, and other marginalized groups. These populations commonly face significant challenges in adhering to daily HIV treatment regimens. Conducting clinical trials of long-acting treatment targeting specific unmet medical needs of these populations can improve understanding of optimal care approaches, broaden the indication for use of long-acting products, and inform treatment guidelines, all of which can influence reimbursement and access policies. Innovative trial designs and programmatic implementation can improve inclusivity for long-acting therapy. This article summarizes discussions of a multistakeholder workshop on study designs for long-acting HIV treatments.

## Teaser

Clinical trials for long-acting HIV treatments tend to underrepresent marginalized populations and adherence-challenged individuals. This impacts drug approval labels and guidelines and thus can limit access. Innovative approaches, such as single-arm, factorial, and pragmatic trials, are needed for greater inclusivity.

Significant disparities in HIV acquisition risk, access to antiretrovirals (ARVs) for treatment, and viral suppression persist in the United States between racial groups, specific populations, and geographies, among others [1, 2]. Black and Hispanic/Latino gay, bisexual, and other men who have sex with men (MSM) and Black heterosexual women account for the majority of new HIV cases [2]. Infections among trans and gender-diverse people have increased by about one-quarter between 2018 and 2022 [2]. In 2022, 61% of Black MSM living with HIV were virally suppressed, compared to 73% of White MSM [1]. Among those whose infection is attributed to injection drug use, only 55% were virally suppressed, with less than half of Black and Hispanic/Latino men infected through injection drug use being virally suppressed [1]. The US Centers for Disease Control and Prevention (CDC) identifies 48 counties (plus Washington, DC, and San Juan, Puerto Rico) that account for more than half of all new HIV infections, and 7 states with a substantial rural burden, with transmission often driven by injection drug use [3]. People who inject drugs (PWID), accounting for 7% of new HIV infections in the United States [2], face systemic barriers to antiretroviral therapy (ART) access and adherence support; they are more likely to experience homelessness, face untreated mental health challenges, have less engagement with medical services, and have a higher likelihood of incarceration [4].

Long-acting ART expands existing options for HIV treatment. Long-acting agents have been developed in other fields—such as for contraception, antipsychotics, and opioid or alcohol use disorders—with the aim of increasing use in patients who cannot or prefer not to take daily pills [5, 6]. Based on the results of 2 large phase 3 clinical trials [7, 8], the US Food and Drug Administration (FDA) approved injectable cabotegravir and rilpivirine (CAB/RPV) for the treatment of HIV-1 for monthly use in adults who are virologically suppressed on an existing ART regimen [9]. Results of a third, large, phase 3 clinical trial were the basis for expanding to bimonthly injections at higher doses [10, 11]. Additionally, lenacapavir, a subcutaneous injection every 6 months, is approved by the FDA for use in combination with other ARVs in treatment-experienced adults [12, 13]. Other agents for long-acting ART, such as long-acting oral nucleoside reverse transcriptase translocation inhibitors [14, 15] and an integrase strand transfer inhibitor [16], as well as broadly neutralizing antibodies [17], are in development.

However, the trials that led to the regulatory approval of long-acting ART regimens underrepresented many of the populations most affected by HIV and least likely to be virally suppressed. This includes marginalized people such as trans and gender-diverse people, PWID, and those with housing insecurity. Moreover, the clinical trials that informed the FDA approval of long-acting CAB/RPV (FLAIR, ATLAS, and ATLAS-2 M) [7, 8, 10, 11], as well as an ongoing trial in 3 African countries (CARES) [18], required participants to be virologically suppressed on oral ART before the switch to long-acting ART, thereby limiting the number of patients with challenges to oral therapy. Although emerging data, such as preliminary results of the LATITUDE trial [19], suggest that CAB/RPV is appropriate for people with adherence challenges, innovation in clinical trial designs is needed for broader inclusivity and to further the impact of new long-acting therapies on the HIV epidemic.

In this article, we summarize presentations and discussions from a multistakeholder meeting held in November 2022 on clinical trial design for HIV treatment, with a focus on long-acting ART. The objectives of the meeting were to (1) review the inclusion of diverse populations in clinical trials of novel ART products, (2) discuss how regulatory decisions, guidelines, and reimbursement policies can facilitate or hinder access to new ARVs, and (3) consider innovative study designs to increase clinical trial inclusivity, particularly of marginalized and adherence-challenged populations. The meeting was convened by the Forum for Collaborative Research, a public–private partnership based at the University of California, Berkeley, and included representatives from the pharmaceutical industry, academic researchers, community and patient advocates, clinicians, and federal agencies (Department of Housing and Urban Development; Health Resources and Services Administration; FDA; National Institutes of Health Office of AIDS Research; National Institute of Allergy and Infectious Disease). Discussions during the meeting focused primarily on the HIV epidemic in the United States but many insights are relevant for the control of HIV in other regions. Details on the workshop, including a list of participants, can be found online (https://forumresearch.org/hiv-forum/treatment/hiv-treatment-meetings/1790-expanding-inclusion-for-long-acting-hiv-treatment-trials-workshop-11102022/).

## LACK OF INCLUSIVITY IN CLINICAL TRIALS ON HIV

Clinical trials provide evidence for the efficacy and safety of new pharmaceutical agents. Including a broad range of participants in clinical trials, including those historically underrepresented, is needed to provide evidence for efficacy in specific populations, reflect the population affected by the condition, and, ultimately, ensure that all communities that could benefit from the product will have access to it. Women, especially Black women, tend to be underrepresented in phase 3 clinical trials on HIV [20, 21]. Additional populations that may be underrepresented in large phase 3 studies include racial minorities, pregnant and breastfeeding people, adolescents, trans and gender-diverse people, older adults, the unhoused, PWID, and people with serious or untreated mental health conditions. This underrepresentation has also been found in non-randomized implementation studies [22]. Furthermore, although the CDC has identified rural hot spots in the HIV epidemic, clinical trials in those areas are rare. The FDA has recently highlighted the need for expanded diversity in clinical trials and released guidance to trial sponsors regarding increasing representation [23].

Experience in other health areas highlights that broader inclusivity is feasible. Although recent ART efficacy studies did not specifically exclude PWID, they remain underrepresented because studies have generally excluded those for whon sub-optimal adherence to dosing may result in safety concerns or who were deemed to be of increased risk of loss to follow-up, which could jeopardize efficacy analyses. In contrast, previous major clinical trials of direct-acting antivirals to cure hepatitis C virus (HCV) have not excluded people who use drugs when studying antiviral effects [24]. Other studies in HCV research have successfully included PWID to examine both drug–drug interactions between ARVs and injection drugs, as well as specific adherence challenges faced by PWID [25]. These HCV studies among PWID have been critical to direct-acting antiviral regimens label expansion and removal of restrictions based on ongoing drug use [26]. Of note, the ongoing PURPOSE 4 study evaluates the pharmacokinetics and safety of the long-acting injectable lenacapavir for HIV prevention among PWID [27].

Similarly, although long-acting agents are new to the HIV treatment field, they have been commonly used in psychiatry [6, 28, 29]. Long-acting psychiatric medications have been shown to be highly cost-effective and well tolerated, with clinical trials demonstrating superior or noninferior efficacy to their oral counterparts. Clinical trial research in psychiatry offers examples of how to include people with mental health challenges; moreover, the inclusion of these populations in HIV clinical trials offers an opportunity to better understand HIV treatment outcomes in these patient populations and how to provide patient-centered care that addresses multiple health issues.

## CLINICAL RESEARCH AND THE ROAD TO ACCESS

Evidence generated by registrational clinical trials is the cornerstone of regulatory approval processes and, therefore, access to new treatment options. The drug label (indication for use), treatment guidelines, and reimbursement policies for pharmaceuticals are all informed by clinical trials, and, to different extents, evidence generated after approval. The inclusion of diverse populations in clinical trials and other studies therefore impacts the inclusion of population-specific considerations in labels and guidelines and can facilitate or hinder access to new ART options.

### US FDA Approval and Drug Labels

The role of the US FDA is to review the quality, safety, and efficacy of candidate drugs. The drug label is determined based on the evidence submitted to the FDA, which does not necessarily include every premarketing trial of the candidate drug, such as studies not conducted by the drug developer and not submitted in the new drug application. Moreover, not all postmarketing trial results are submitted to the FDA for label updates because not all trials meet the regulatory standards to enable a label change [30]. Although the FDA encourages study sponsors to investigate their products in specific subgroups, the agency cannot mandate they do so (with exceptions for safety concerns and the pediatric population). The FDA does, however, evaluate the efficacy of ARVs depending on the study population's previous history with ARV use. Virologic endpoints are evaluated on whether the population has not been treated before, has been previously treated with some degree of drug resistance, or has widespread resistance to various ARVs [30].

Once a drug is approved, it is allowed to be marketed based on the drug label. Inclusion or omission of language around use of a drug in a specific population in the label may facilitate access to the drug or create barriers, although healthcare providers in the United States have latitude to prescribe a medication “off-label” to a population not explicitly indicated in the drug's label. New data are required to support changes in the label, such as expansion of indication to additional populations not included in the original submission. Such information is typically provided by the sponsor with a request to change the product label. Examples of these label changes in HIV treatment include expansion of indication from those not previously treated to both those previously untreated and those with treatment experience, such as with the dual ART combination of dolutegravir/lamivudine (DTG/3TC) [31].

### HIV Treatment Guidelines

In contrast to FDA labeling, HIV treatment guidelines are based on all available evidence for ARVs. One example is the US Department of Health and Human Services HIV Clinical Guidelines [32], developed by a multidisciplinary panel of experts convened by the National Institutes of Health (NIH) to synthesize currently available information into practical guidelines on HIV treatment for clinicians. The guidelines are updated regularly.

When evaluating evidence, the guidelines panel ranks evidence based on both the strength of the recommendation and the type of evidence informing the recommendation. Although randomized controlled trials (RCTs) are the most robust source to inform HIV treatment guidelines, the guidelines are not restricted to label indication, and the entirety of evidence available informs the panel's recommendations for treatment of people with HIV, including for specific populations, even where these are not included in the drug's label. Guidelines have impact on clinical practice, including prescribing behavior, and reimbursement policies, and therefore can have significant impact on the availability of, access to, and use of products.

### Policies for Reimbursement of Long-acting ARVs

Reimbursement for any HIV medication in the United States is highly complex. Various funding programs exist to allow people living with HIV access to treatments. These include private insurance, public insurance (Medicare and Medicaid), Ryan White funding, and manufacturer rebates. There can be overlap in the programmatic coverage of individuals in these programs, and each program may classify each long-acting ARV differently, leading to varied access across populations based on which program and reimbursement path is available and used.

Although a drug label spells out the population for which an ARV is indicated, the FDA does not consider or comment on drug cost in its evaluation process, nor does it engage in discussion of policies on insurance coverage or reimbursement for health products. Therefore, the label does not specify whether a drug will be reimbursed. Policies of various payers determine what information is considered to require reimbursement of an ARV. Depending on the payer, HIV treatment guidelines may hold more weight in determining the ability of an individual to access long-acting ARVs. Hence, including considerations for specific populations in guidelines is important for access.

## PROMISING APPROACHES IN HIV TREATMENT TRIALS

There are many innovative approaches to clinical trial design and study implementation that can broaden inclusivity, generate data to inform drug labels and treatment guidelines, and contribute to the body of evidence to support a drug's use in diverse populations.

### Single-arm and Investigator-initiated Trials and Commercial-partnerships

Single-arm trials, in which all trial participants receive the study intervention, are particularly important for groups for which randomization is not feasible or when using a placebo-controlled design might be challenging. Although these study designs may not meet the regulatory standards for evaluating the efficacy of an investigational product, they allow for generating data on dosing, safety, and acceptability of products, including among individuals who were unable to participate in RCTs. Single-arm trials also allow for evaluating associations between product use and clinical outcomes, and effectiveness can be evaluated through comparisons across multiple time points (eg, baseline vs follow-up). Because there is no control arm for comparison, single-arm trials require robust predefined thresholds to determine whether a treatment failed [33]. One example of a single-arm open-label trial is the ongoing MOCHA trial in which virally suppressed adolescents aged 12 to 17 years switched from an oral ART regimen to CAB/RPV and found no virologic failure through week 24 of study follow-up [34]. Moreover, the ILANA study is an ongoing single-arm study on CAB/RPV in virally suppressed adults in the United Kingdom [35]. The study explicitly aims to recruit underrepresented populations by capping participation of male and White participants at 50% of all study participants and participation of adults younger than 50 years of age at 70%. This strategy of setting recruitment targets could be integrated into clinical trials to ensure adequate representation of different populations. For instance, PURPOSE 2, an ongoing phase 3 clinical trial on the long-acting injectable lenacapavir for HIV prevention, established a goal of recruiting 50% Black and 20% Hispanic/Latinx MSM in the United States as well as 20% transgender women across global study sites [36].

Investigator-initiated studies are studies initiated by nonpharmaceutical company researchers that commonly represent partnerships with commercial companies. They provide the advantage of giving significant leeway to generate hypotheses, investigate unique questions, and conduct research within a specific area of expertise or focus [37]. An example of an investigator-initiated single-arm study is a study at Ward 86 in San Francisco. The study investigated long-acting CAB/RPV use in individuals with and without adherence challenges and found that 98% of study participants were virologically suppressed after 26 weeks [38]. Further analyses found that, among those initiating CAB/RPV without being virally suppressed, 93% were virally suppressed at week 48 of study follow-up [39]. The study, therefore, supports further research into the use of CAB/RPV and other long-acting ART in adherence-challenged, nonvirologically suppressed populations (including, potentially, individuals who have never received ART). The study also demonstrated the feasibility of integrating long-acting treatments for HIV and mental health services [38]. Investigator-initiated studies are helpful to inform treatment guidelines but are generally not used in the initial labeling of a pharmaceutical agent. Although information from investigator-initiated studies can be used to inform labeling, their submission to the regulatory authority is incumbent on the commercial sponsor.

### Pilot Studies to Inform RCTs

Pilot studies allow for investigating highly specific questions compared to larger randomized clinical trials, serving as proof of concept to invest resources into a larger, more complex trial. One example is the investigation of DTG/3TC. Initially, FDA reviewers had concerns with proposals to investigate DTG/3TC in adults who have never received treatment, particularly with higher viral load levels, because of an absence of additional supporting data, concerns about the durability of only 2 active agents, and potential for resistance of a 2-drug regimen in previously untreated adults. Two pilot studies, PADDLE and AIDS Clinical Trial Group (ACTG) 5353, investigated individuals with baseline HIV RNA levels of less than 100 000 copies/mL and 500 000 copies/mL, respectively [40, 41]. The favorable results from these pilot studies led to an amendment of the trial protocols for the GEMINI-1 and GEMINI-2 studies, greatly increasing the HIV RNA threshold for exclusion [42]. In turn, these phase 3 studies demonstrated non-inferior efficacy of DTG/3TC to a standard 3-drug regimen and led to FDA approval for adults who have never received treatment, and the label was later amended to include virally suppressed adults.

Pilot studies are unlikely to be comprehensive enough to change drug labeling or treatment guidelines for long-acting ART. Still, they could provide early evidence and inform the inclusion criteria, including underrepresented populations, for future RCTs, whose results could hold more weight to influence labeling and guidelines.

### Bridging Efficacy and Implementation Research

Studies that bridge the gap between efficacy trials and implementation research will be vital to ensuring access to long-acting HIV treatments. For instance, factorial trials involve study participants randomized to various combinations of interventions. Trial participants might be randomized to treatment A, treatment B, both treatment A and B, or neither treatment A nor B. Factorial trials are efficient study designs as they can investigate the efficacy of various interventions simultaneously. They are also particularly useful for determining the potential additive or synergistic effects of combination treatment. In the context of HIV treatment research, the factorial approach could test different treatment support strategies, such as psychosocial support and counseling [43], the provision of harm reduction and housing services for unstably housed people with HIV [44], or assertive community treatment in which comprehensive care is delivered in community settings [45]. These strategies could improve the inclusion of underrepresented populations in studies as they address complex health needs and remove barriers to accessing care. Such a design would preserve randomization and provide valuable data for treatment implementation strategies and guidelines.

Pragmatic trials inform clinical and policy decision-making by providing evidence of the effectiveness of an intervention in real-world settings. Pragmatic trials often offer more opportunities for investigators to broaden study inclusion criteria and assess real-world factors that may influence uptake of or adherence to a drug [46]. As such, pragmatic studies can bridge efficacy and implementation research, particularly following FDA approval. Although pragmatic studies are unlikely to be used for initial registration of a new molecular entity, their results can contribute to the body of evidence that informs HIV treatment guidelines and can better inform clinical care in specific populations. The ILANA study, for instance, evaluates CAB/RPV implementation in real-world clinical settings in the United Kingdom, including the effectiveness of clinic-based delivery compared with community-based delivery by initially only offering clinic injections and, after 6 months, offering a choice of in-clinic and community injections [35]. Community-based delivery of HIV treatment in particular has the potential to remove barriers to care and improve inclusion of marginalized populations.

### Leveraging Collaborative Networks

The HIV field highlights the usefulness of collaborative networks in strengthening research. The ACTG, International Maternal Pediatric Adolescent AIDS Clinical Trials Network, HIV Prevention Trials Network, and HIV Vaccine Trials Network are NIH-funded collaborative networks aiming to reduce barriers to cooperation and improve efficiency in HIV prevention and treatment clinical trials. These networks can be leveraged for studies that include participants from underrepresented populations. For instance, ACTG A5359 (LATITUDE) enrolled individuals previously nonadherent to oral ART regimens and compared injectable CAB/RPV and standard-of-care oral ART regimen, with preliminary findings suggesting a lower incidence of virologic failure among those receiving injectable ART in this population of people with adherence challenges [19]. Such collaborative networks exist in a range of countries, allowing for cross-learning. For instance, in France, the National Agency for Research on AIDS and Viral Hepatitis facilitates research across a large number of centers, including on innovative HIV treatment approaches [47]. Similarly, in Switzerland, the long-running Swiss HIV Cohort Study has implemented a real-world multicenter study on CAB/RPV [48].

However, despite the immense contributions of these networks, their recruitment reach is often confined to trial participants living near large academic centers. Of the 7 US states identified by the CDC as having substantial rural epidemics, only 2 contain trial sites within a major national HIV treatment trial network, and both of those sites are in major metropolitan areas [3, 49]. Given the evolving nature of the HIV epidemic in the United States, investment in innovative trials in regions outside of the geographic bounds of traditional clinical trial centers is needed. To broaden the geographic reach of NIH funding for clinical trials and other biomedical research, the NIH has established the Institutional Development Awards (IDeA) Program [50]. IDeA-eligible states are strong candidate areas for innovative clinical trials. For example, the IDeA program-funded West Virginia University Clinical and Translational Science Institute has invested in a mobile clinical trials unit. This retrofitted van can travel between rural communities to increase the site's ability to participate in clinical research [51]. Further, the institute engages the Practice-Based Research Networks throughout the state to widen the reach to rural populations [52]. These efforts greatly extend the benefit of clinical trial participation to people who traditionally are unable to participate.

## CONCLUSION

The HIV epidemic in the United States is evolving, as is our ability to use long-acting treatments to combat it. There is a need to explore innovative ways to ensure that those who tend to be underrepresented in clinical trials and carry a disproportionate burden of the epidemic are not left behind in efforts to eliminate HIV. Innovative and collaborative approaches to trials of long-acting treatment options with diverse study populations can generate data to influence product labeling and guidelines to include diverse and underrepresented populations, thus expanding access to long-acting HIV treatments for marginalized populations and those with adherence challenges. To end the HIV epidemic in the United States, the inclusion of these populations is imperative and requires resources, dedication, and innovation. However, clinical trial inclusivity is only one important step to improve access to HIV treatments for marginalized populations. It is equally important to address the various individual, social, and structural barriers to accessing healthcare that many individuals from these populations face [53, 54]. This includes the lack of universal healthcare in the United States, with 26 million people, or 7.9% of the total population, not having health insurance [55], as well as lack of affordable housing, limited harm reduction programs, stigma and discrimination (including in the healthcare system), and punitive laws and policies, among other factors. Many of these topics were beyond the scope of the meeting that formed the basis of this article, and the Forum for Collaborative Research continues to convene further multistakeholder meetings to address diverse issues around access to the latest HIV treatment and prevention options in the US and globally.

## Notes

***Acknowledgments.*** We thank all participants of the meeting that led to the development of this manuscript. A list of participants is available online: https://forumresearch.org/hiv-forum/treatment/hiv-treatment-meetings/1790-expanding-inclusion-for-long-acting-hiv-treatment-trials-workshop-11102022/.

***Author Contributions.*** N.A.M. and N.A.E. wrote the first draft of this article. R.S. finalized the draft. All authors contributed to the discussions underlying this article, contributed to the writing of the manuscript, and reviewed the final draft.

***Financial support*.** This work was supported by the HIV Forum of the Forum for Collaborative Research, which receives unrestricted grants from Gilead Sciences, Merck Sharp & Dohme LLC, a subsidiary of Merck & Co., Inc., Rahway, NJ, USA, and ViiV Healthcare.
